# Assessment of Diabetic Foot Prevention by Nurses

**DOI:** 10.3390/nursrep13010008

**Published:** 2023-01-09

**Authors:** Sonia Hidalgo-Ruiz, María del Valle Ramírez-Durán, Belinda Basilio-Fernández, Pilar Alfageme-García, Juan Fabregat-Fernández, Víctor Manuel Jiménez-Cano, Maria Zoraida Clavijo-Chamorro, Adela Gomez-Luque

**Affiliations:** 1 Department of Nursing, University of Extremadura, 10600 Plasencia, Spain; 2 Department of Nursing, Faculty of Nursing and Occupational Therapy, University of Extremadura, 10004 Cáceres, Spain

**Keywords:** diabetic foot, prevention, therapeutical education

## Abstract

Diabetic foot is a severe complication of diabetes, with serious consequences such as amputations and high mortality rates as well as elevated economic costs. To evaluate whether or not nursing staff follow the recommendations of national and international organizations regarding diabetic foot prevention, a cross-sectional and observational descriptive study was carried out using an ad hoc self-administered questionnaire validated by seven experts, with a Cronbach’s alpha of 0.731. Of the total 164 participants, 157 met the inclusion criteria. Findings showed that 96.58% asked their patients to remove their footwear, 78.34% performed thorough examinations, and 80.25% assessed the risk of developing diabetic foot. Participants educated their patients in self-care and evaluated skills related to diabetic foot control either frequently (84.07%) or very frequently (62.42%), and only 19.11% of them carried out group activity workshops. Significant statistical differences were found in the performance of activities in the groups by participant age intervals, whether working in primary health care or a hospital, having specific training, and the participant’s DM patient ratio. We obtained high percentages of compliance in the assessed activities in comparison to other studies. Nevertheless, we believe it is necessary to encourage screening in specialized care, skills testing, and the implementation of educational group activities and workshops.

## 1. Introduction

According to the International Diabetes Federation (IDF), the prevalence of Diabetes Mellitus (DM) is estimated at 10.5% in the world and 10.3% in Spain for the population aged 20 to 79 years old. Therefore, at present, 537 million people have DM in the world, and more than 5.1 million are in Spain. Given the IDF forecasts for 2045, the future is not at all encouraging, with figures soaring up to 783 million people with DM in the world and 69 million in Europe [1].

The estimated lifetime risk of a person with DM developing a diabetic foot (DF) is between 15% [2,3] and 25% [4], potentially rising to 34% [5]. Thus, according to current IDF figures, up to 282 million people worldwide and more than 1.6 million in Spain could develop DF disease during their lifetime [1].

The direct and indirect costs associated with the treatment of DF ulcers average EUR 10,000, rising to EUR 16,835 if infection and arterial disease coexist [6]. This expenditure is mainly at the expense of increased hospital stays which can account for 14% of the total hospitalization costs of people with DM [7].

We cannot forget the social costs of the DF ulcer and one of its complications, amputation, which occurs in 14–20% of cases [8], both of which are very disabling conditions that will prevent the patient from having a normal working life and will involve the family in their care. In addition, the risk of death is 2.5 times higher 5 years after the injury [5,9] and 2 times higher 10 years later [10].

Considering the high prevalence and mortality and the elevated economic and social cost of DF, there is an urgent need to improve healthcare systems, especially as 49–85% of all DF problems are preventable if appropriate measures are put in place [2]. Institutions such as the American Diabetes Association and the International Diabetic Foot Task Force consider these prevention tools essential [11,12].

One of the basic pillars of treatment and prevention of complications in any chronic disease is therapeutical education. This must be based on the detection of risk and is essential for establishing preventive measures and tailored care plans for patients. In this regard, the role of nursing in the therapeutical education of diabetes and diabetic foot is key to achieving success in the prevention of ulcers and amputations.

Raising awareness regarding diabetic foot among the administration, professionals, and patients is paramount, via prevention programs in what are called “complex educational interventions” [13].

The aim of this study is to assess the caring path taken by RNs regarding diabetic foot prevention, focusing on barefoot exploration, inspection, and educational activities in order to detect weaknesses that will ease proposals for improvement. 

As a conclusion of the study, we will emphasize the evaluation of patients’ skills and the implementation of practical group education workshops.

## 2. Materials and Methods

A descriptive, cross-sectional, observational study was carried out using a self-administered “ad hoc” questionnaire. This study was carried out from January to November 2019.

The study was conducted according to the guidelines of the Declaration of Helsinki and has the approval of the Bioethics Committee of the University of Extremadura with the number 147/2022 approved on the date 28 September 2022.

### 2.1. Scope, Study Subjects, and Eligibility Criteria

The scope of the study was national (it was carried out in Spain), and the subjects (women and men) had to meet the criterion of being active practicing nurses in Spain.

The sample size was based on convenience sampling for the selection of participants. The initial total number of participants was 164, out of which 6 were excluded because they were not working in Spain and 1 for not being a nursing professional. Thus, the final sample was 157 participants.

The questionnaires were completed in 10 min and no feedback was provided to the participants. Anonymity and confidentiality were assured to all participants through the presentation of informed consent at the beginning of the questionnaire. In addition, participants were informed that the data would be disclosed for research purposes.

### 2.2. Questionnaire Development Process

To develop the “ad hoc” questionnaire, we took as a reference those carried out by other authors [14,15,16] and introduced items taking into account the recommendations for the management of DF proposed by various international organizations such as the American Diabetes Association (ADA), the International Working Group of Diabetes Foot (IWGDF), the National Institute for Health and Care Excellence (NICE), the International Diabetes Federation (IDF) [1,11,12,17], and national organizations such as the General Council of Podiatrists’ Associations (CGCOP), the Spanish Society of Angiology and Vascular Surgery (SEACV), and the National Conference on Ulcers of the Lower Extremity Conference (CONUEI) [18,19].

The validation of the questionnaire followed very strict steps. First, the questionnaire that was prepared based on official agencies was evaluated by two university nursing professors and a statistician, incorporating their proposed changes into the questionnaire.

Second, the questionnaire was reviewed by five experts in the field—two doctors and three nurses—who acted as validators. These experts were provided with a summary of the project containing the objectives, study design, scope, and subjects to be studied.

Once the questionnaire had been analysed, the experts had three questions to answer:

1. Do you think that all the items in the questionnaire can be understood by the nursing staff? If not, which items are not well understood? How would you re-word them?
2. Do you consider the questionnaire suitable for the goals of the study? If not, why is it so?
3. Proposals for improvement of the questionnaire: Would you remove any item? Would you add any item?

Questions 1 and 2 obtained 100% affirmative responses, which assured us that the questionnaire was understandable and appropriate. Among the proposals for improvement in response to question 3, the following were taken into account:

- Include age ranges below 35 years of age;
- Distinguish between patients with type 1 diabetes mellitus (T1DM) and type 2 diabetes mellitus (T2DM);
- Ask about patients with DF ulcers treated in the last 3 months;
- Use the Likert scale in some of the questions.

The proposals for improvement were introduced in the final questionnaire form. Lastly, a pilot screening was performed by 15 nurses to detect possible errors. Once amended, the final version of the questionnaire was launched by email from diabetes institutions in Spain in addition to social networks.

The variables that were finally analysed were:

*Sociodemographic characteristics:*

- Gender: male or female.
- Stratified sampling. Age: <26; 26–35; 36–45; 46–55; 56–65; or >65.
- Years in active service: <6; 6–10; 11–20; 21–30; 31–40; or >40.
- Workplace: primary healthcare; hospital; nursing homes; diabetic foot care units; TVN units; diabetes mellitus educators; dialysis; several centres; or others.
- Specific training: none; health system; university laboratories; self-financed; Nurse Resident Intern; health system + university laboratories; health system + self-financed; university laboratories + self-financed; health system + university laboratories + self-financed; or university.
- Complementary training: podiatry; Nurse Resident Intern; or podiatry + Nurse Resident Intern.
- Quotas of patients with T1DM or T2DM: 0; 1–50; 51–100; 101–200; 201–300; 301–500; or >500.
- Ulcers treated in the last 3 months: 0; 1–10; 11–25; 26–50; 51–100; 101–200; or >200.

*Prevention activities:*

- Barefoot exploration: never; no response; according to risk; with symptoms; four times/year; three times/year; two times/year; one time/year; + than once /month; or all visits.
- Inspection: analysing the frequency that the total number of participants inspect or evaluate certain parameters such as footwear, socks, temperature, pain and colour of the feet, the existence of oedema, keratopathies, onychopathies, and foot deformities; yes; no.
- Risk assessment: yes or no.
- Therapeutic education.
  ▪ Self-care education: this refers to the frequency that topics such as washing, hygiene, drying and hydration, inspection and recognition of lesions, nail clipping, choice of footwear and socks, and education on DM, in general, are addressed; rare; occasional; frequently; very frequently; or never.
  ▪ Checking skills, knowledge, and environment: rare; occasional; frequently; very frequently; or never.
  ▪ Conducts workshops: yes or no.
  ▪ Reasons for not conducting workshop: no response; time; training; type of patient; no service; individual; resources; time + individual; or time + resources.

### 2.3. Statistical Analysis

The statistical evaluation of the results was carried out with the SPSS v.28.0.1 statistical package (SPSS, Chicago, IL, USA) for Windows, setting the statistical significance at a value of *p* < 0.05, which represents 95% confidence.

To test the internal reliability of the questionnaire, Cronbach’s alpha was calculated. Most of the values obtained were expressed as percentages, using the Chi2 test to establish the relationship between the characteristics of the population and the different activities evaluated.

## 3. Results

### 3.1. Description of the Population

A total of 157 nurses participated in the study. The socio-demographic characteristics of the participants are shown in Table 1.

#### 3.1.1. Gender and Age

Of the 157 participants, 120 were female and 37 were male, representing 76.43% and 23.57%, respectively.

The distribution of the sample by age range shows that most of the participants were aged between 36 and 45, followed closely by the 46–55 year old range. Thus, 84.08% of the subjects were under the age of 55 years old, concentrating the population in the so-called stage of mature adulthood. The interval of 36–45 years is also the most predominant in both men and women when analysed separately.

#### 3.1.2. Years in Active Service

Regarding years in active service, the intervals of 21 to 30 years and more than 40 years in active service represent the highest and lowest percentages respectively. A total of 73.25% of the sampled population has more than 10 years of professional experience.

#### 3.1.3. Workplace

Participation included all the Autonomous Communities of Spain with the exception of the Canary Islands, Cantabria, and Navarre. The greatest participation by the Autonomous Communities was in Extremadura, representing 40.76% of the sample. The distribution of participants by province shows a great response in the provinces of Badajoz and Caceres, with 36 and 28 participants, respectively.

Regarding the level of care, 49.68% of the participants worked in primary care and 19.75% in hospital care, representing 69.43% of the sample. For the remaining locations, 10.19% worked in nursing homes and 8.28% combined their work in various centres, usually combining nursing homes with primary care or hospital care, or primary care with hospital care. The item “others” include centres such as penitentiary institutions, drug addiction rehabilitation facilities, mental health units, A&E, or pain units.

#### 3.1.4. Training

Analysing the specific training on DF of the participants, we found that 15.92% of they, had no training in this subject, 30.57% received self-financed training (SF), and 19.11% had training supported by the public health system (HS).

Specific training was received only via laboratories (Lab), while obtaining university degrees, or by the Nurse Resident Intern Program (NRI), representing 5.73%, 2.55%, and 1.27%, respectively.

In addition, 11 had a diploma/graduate degree in podiatry and 10 had been an NRI, representing 6.37% and 12.74%, respectively. Only one NRI was also a podiatrist, representing 0.64% of the sample.

#### 3.1.5. Quotas of Patients and DF Treated

As can be seen in Table 2, most of the participants who responded to the question about their quota had a total quota (with and without DM) of more than 500 patients (67.72%, n = 127).

Regarding the quota of patients with DM, most participants had a quota of less than 50 (47.14%, n = 70), as occurs among the quota of patients with T1DM (84.13%, n = 63) and T2DM (49.18%, n = 61) when both are analysed separately.

Table 3 shows the ulcers that the participants had treated in the last 3 months. A total of 29.30% of the sample had not treated any ulcer and the most predominant interval was 1 to 10 ulcers, representing 58.60%.

### 3.2. Prevention Activities: Barefoot Exploration, Inspection, Risk Assessment, and Therapeutic Education

The data on the inspection and risk assessment of the total number of nursing staff who participated in the study are presented below (Table 4).

#### 3.2.1. Barefoot Exploration

Of the total number of participants (n = 157), 36.31% of them explored their DM patients’ barefoot feet at all visits. A total of 14.01% do so when symptoms are present and 8.28% according to the risk detected in the examination. The number of participants who never practiced barefoot exploration was 3.18% (Table 4).

#### 3.2.2. Inspection

Analysing the frequency that the total number of participants (n = 157) inspect or assess certain parameters, such as footwear, socks, temperature, pain, changes in skin colour on the feet, presence of oedema, keratopathies, onychopathies, and foot deformities, 78.34% performed inspections compared to 21.66% who did not, equivalent to 123 and 34 participants, respectively (Table 4).

#### 3.2.3. Risk Assessment

Of the total sample (n = 157), 31 participants did not perform a DF risk assessment and 126 did, this represents 19.75% and 80.25% of participants, respectively (Table 4).

#### 3.2.4. Therapeutical Education

- Education in self-care: this refers to the frequency that topics such as washing, hygiene, drying and moisturizing, inspection and detection of injuries, nail clipping, choice of footwear and socks, and DM education, in general, are addressed. A total of 49.04% of the respondents addressed all of these topics very frequently and 35.03% frequently. The percentages referring to occasionally, rarely, and never, did not reach 16% of the sample (Table 4).
- Checking skills, knowledge, and environment: the percentage of participants who assessed the patient’s mobility, visual acuity, knowledge, skills, care environment, and social situation was 94.27%. The frequency in which they do so varies from 10.19% who assess it rarely, to 42.04% who assess it frequently (Table 4).
- Conducting workshops: 30 participants conducted practical workshops on foot care with their patients with DM, which represents 19.11% of the sample. This means that 80.99% do not carry out workshops (n = 127, 80.99%).
  Participants were asked why they did not conduct practical workshops. The responses are shown in Table 5, where we can see that 27.56% of the cases claimed a lack of time, 13.39% did not have the resources and tools, and 12.60% did not work in the relevant service. The type of patient (with dementia, for example) was the reason for 11.02% of cases, and carrying out education on an individual basis instead of the form of workshops was 7.09% of cases. A total of 0.79% of cases indicated they did not have sufficient training and 25.20% did not answer.

### 3.3. Bivariable Analysis

Table 6 shows the results of the Chi-squared (χ^2^) test and its significance, analysing the different population groups in reference to the performance of the activities evaluated. The correlations between the variables result in a Cronbach’s alpha of 0.731. Sex, years in active service, or being an NRI did not have a significant influence on the performance of the activities. The groups in which we found significant differences are shown below.

According to the age intervals, the differences are produced at the expense of a higher percentage of barefoot exploration (97.56%) and inspecting (78.05%) in the interval of 46–55 years old, and a lower percentage of barefoot exploration in the group of 26 to 35 years old (75%) and inspecting in the group under 26 years (42.86%).

The workplace often conditioned the performance of the examination, with 89.05% in primary care, compared to 64.7% in a hospital.

Participants with specific training in DF performed a higher percentage of examinations than those without, 86.05% compared to 60%.

Participants with quotas above 300 patients with DM performed 100% of the examinations; the lowest quota was 1–50 patients with 69.7% of case examinations. Those with a quota of more than 200 patients with DM and those who have treated between 51–100 ulcers in the last 3 months classified 100% of DF ulcers. Participants with a quota of 1–50 patients with DM and with more than 100 ulcers treated were the least likely to classify DF, with 54.4% and 0%, respectively.

## 4. Discussion

The bibliography consulted for this work recommends that at least one annual diabetic foot and risk screening should be performed [11,12,18]. In our sample, up to 80.25% of the participants’ patients were screened for DF risk, 75.16% for patients with T1DM, and the same figure for T2DM. There are significant differences depending on the number of patients with DM, with 100% of the participants being screened in groups of more than 300 patients.

The percentages shown are considerably higher than those reflected in the study by Bernal et al., with 32 patients in a haemodialysis unit, in which only 22.56% were assessed for risk, despite the fact that 6.45% had DF at that time and 34.48% were at high risk of developing it [20]. Galiano et al. estimated the annual assessment of the risk of DF and amputations, carried out by RNs in patients with T2DM, at 78% [21], a percentage slightly higher than ours.

The study by Ledesma et al., which evaluated 192 consultations in health care centres and 18 in hospitals in Malaga, showed that in primary care, only 54% of the clinical record templates included annual foot examinations. In hospital care, the results are higher, reaching 67% [22]. Our results are higher in primary care, with 89.7%, but in hospital care, they are lower, 64.5%. The chi-squared test indicated that there are significant differences between these percentages. We attribute these differences to the fact that DF is usually seen in specialized care when it is already established and usually with the presence of complications.

In Extremadura, Basilio’s work in 2015 reflects low rates of professionals carrying out DF screening. Specifically, the eight nurses included in an educational intervention program in DF did not even perform 16% of the expected examinations according to their quota of patients with T2DM, despite having received specific training in this regard [23]. In the survey carried out by Martínez in 2016, with the participation of 228 nurses from the Canary Islands, it was estimated that 29% never performed a neuropathy assessment and only 11% always performed it [16].

The study with the lowest figures was performed by Cid et al. in 2017, carried out on 139 patients with T1DM, in which only 3.6% of medical records showed at least one foot examination per year, explaining the low results as a consequence of the lack of specific protocols and clinical records [24].

In our study, we emphasized not only the general examination but also the inspection of the bare feet, an act closely related to the act of removing the patient’s shoes. Until 2018, the ADA indicated the need to inspect the feet of people with DM at each visit [25]. However, in 2019, this criterion was qualified by recommending that inspection at each visit should only be carried out on patients with diabetic neuropathy and a history of ulceration or amputation [26]. Additionally, in 2022, the term “diabetic neuropathy” is no longer used, having been replaced by patients with “evidences of sensory loss” [11]. 

In our study, 36.31% of the participants performed barefoot examinations on their patients at all visits. Furthermore, we found that up to 96.58% of respondents (n = 146) did it sometimes, with periodicities ranging from once a month to once a year, depending on the level of risk or the presence of signs and symptoms. Regarding the variances found between the different age intervals and the act of barefoot walking, it should be noted that it was significantly more frequent in the 46–55 year age interval. A more in-depth analysis would be necessary to launch explanatory hypotheses.

While analysing both the act of removing the shoes to inspect the feet and the examination, we should be aware that standalone periodic foot examinations do not reduce the risk of ulcers and amputations [3]. In this regard, patient education has been shown to positively influence foot-care knowledge in the short term, reducing ulcerations and amputations, especially in high-risk patients [3,27,28]. The meta-analysis by Renders et al., which considered 41 studies with more than 48,000 patients, concluded that in studies with a positive effect on patient outcomes, education activities were generally included [29].

It is understandable, then, that among the recommendations of the ADA, we found that all patients with DM and, particularly, those at high risk for DF, should receive education on foot self-care [11]. In this regard, our results showed that 84.07% of the participants educated their patients on self-care frequently or very frequently and are in compliance with the ADA´s guidelines. This percentage is considerably higher than the results from Galiano et al. who estimated that 55.6% of the participants carried out foot care education by the nursing staff [21].

Ledesma et al. showed that only 10% of the patient education performed by nurses in primary healthcare was later registered in the patient’s medical records and 28% of the patient education was performed in hospital centres [22]. If we split the sample according to whether they work in primary care or hospitals, there are no significant differences between the two groups, obtaining percentages of 84.62% and 83.87%, respectively, results much higher than those obtained by Ledesma et al. [22].

In our analysis, the percentage of participants checking the patient’s mobility, visual acuity, knowledge, skills, caregiving environment, and social situation frequently or very frequently was 62.42%. We consider this to be a low percentage, especially considering the results of the research by Basilio, 2015, in which only 40% of the sample had all the correct skills for good self-care [23]. It is necessary to focus on checking the skills, knowledge, and abilities of our patients in order to seek greater support from family or caregivers and involve them in the process if necessary.

Despite the fact that, in our study, the percentages of nurses performing the examination, removing the patient’s shoes, and educating in self-care are high, this does not occur with regard to conducting practical workshops, i.e., only 19.11% of the sample carried out practical group workshops. Among the main reasons for not carrying them out, the rest of the participants claimed a lack of time or resources. Worse results were obtained in Basilio (n = 84 T2DM), in which none of them had received group therapeutic education. However, part of the sample referred to having received individual advice on foot self-care [23]. In our study, something similar occurred, since 7.09% of the sample replaced group workshops with individual education.

When analysing the best type of therapeutic education, there is controversy in the literature. Some authors argue that group education has a greater impact on basal blood glucose monitoring, knowledge of DM, and the patient’s quality of life than individual education [30,31]. On the other hand, we find studies such as those of Corbett et al. in which, after three sessions of individual education, an improvement was shown in the acquisition of knowledge about self-care, confidence, and a healthy lifestyle [32]. In the same vein, Vatankhah et al., in their study on individual education conducted with 148 patients, achieved improved patient knowledge, increased motivation, and behavioural changes in foot care after an intervention [33]. The meta-analysis by Dorrestejin et al. reports two randomized clinical trials (RCTs) in which patients receiving individual education reduced their occurrence of ulcers and amputations [34]. Our opinion, as well as of other authors, is that group education should reinforce individual education but not replace it, they should be complementary [23,35,36].

## 5. Conclusions

After analysing all the parameters included in the study, there are significant differences in age intervals that influence whether or not the examination of the patient’s feet takes place, i.e., the workplace, if the participant has specific training, and the quota of patients with DM. 

Our results, in general, show higher percentages with respect to the performance of the activities evaluated, including barefoot examination, inspection, and education in self-care, in comparison with various studies reviewed. However, we believe it is necessary to focus on the weak points, increasing specialized care exploration, assessing the skills of our patients to a greater extent, and, above all, promoting and encouraging practical group education workshops. 

It is necessary to establish specific protocols, plans, and guidelines, to be periodically evaluated, that implement the existing resources to increase the percentages of compliance with the activities proposed by official bodies for the prevention of DF since, ideally, these should be followed 100% of the time.

**Study limitation**: Although the questionnaires were disseminated on pages and forums linked to nursing, being anonymous, we did not have control over who answered them or the ability to resolve any questions. In addition, we did not perform a sample calculation; non-probabilistic convenience sampling was used. Furthermore, the survey does not have a preliminary peer review process in the validation.

## Figures and Tables

**Table 1:** Distribution of nurses according to sociodemographic characteristics. (n = 157).

Characteristics	Value	Frequency (n)	Percentage (%)
Gender	Male	37	23.57
	Female	120	76.43
Age	<26	14	8.92
	26–35	32	20.38
	36–45	45	28.66
	46–55	41	26.11
	56–65	25	15.92
	>65	0	0
Years in active service	<6	26	16.56
	6–10	16	10.19
	11–20	40	25.48
	21–30	41	26.11
	31–40	29	18.47
	>40	5	3.19
Work centre	Primary healthcare	78	49.68
	Hospital	31	19.75
	Nursing homes	16	10.19
	Diabetic foot unit	2	1.27
	TVN units	10	6.37
	Diabetes Mellitus educator	1	0.64
	Dialysis	2	1.27
	Several centres	13	8.28
	Others	4	2.55
Specific training	None	25	15.92
	Health system	30	19.11
	University laboratories (Lab)	9	5.73
	Self-financed (SF)	48	30.57
	Nurse Resident Intern (NRI)	2	1.27
	HS + Lab	4	2.55
	HS + SF	16	10.19
	Lab + SF	2	1.27
	HS + Lab + SF	17	10.87
	University	4	2.55
Complementary training	Podiatry	10	6.37
	NRI	11	12.74
	Podiatry + NRI	1	0.64

**Table 2:** Total patients with T1DM or T2DM (n = 127).

Characteristics of Patients	Range of Patients	Frequency of Participant (n)	Percentage of Participants (%)
Total	0	0	0
	1–50	22	17.32
	51–100	10	7.87
	101–200	4	3.15
	201–300	1	0.79
	301–500	4	3.15
	>500	86	67.72
DM	0	0	0
	1–50	60	47.14
	51–100	20	15.71
	101–200	25	20
	201–300	11	8.57
	301–500	2	1.43
	>500	9	7.14
T1DM	0	10	7.94
	1–50	107	84.13
	51–100	4	3.17
	101–200	0	0
	201–300	4	3.17
	301–500	0	0
	>500	2	1.59
T2DM	0	2	1.64
	1–50	63	49.18
	51–100	25	19.67
	101–200	19	14.75
	201–300	10	8.20
	301–500	4	3.28
	>500	4	3.28

**Table 3:** DF Ulcer in the last three months. (n = 157).

Characteristics	DF Ulcer Range	Frequency in Participant (n)	Percentage in Participants (%)
Ulcers treated in the last 3 months	0	46	29.30
	1–10	92	58.60
	11–25	10	6.37
	26–50	5	3.18
	51–100	2	1.27
	101–200	1	0.64
	>200	1	0.64

**Table 4:** Barefoot exploration, inspection, risk assessment, and therapeutic education. (n = 157).

Interventions	Value	Frequency (n)	Percentage (%)
Barefoot exploration	Never	5	3.18
	No response	11	7.01
	According to risk	13	8.28
	With symptoms	22	14.01
	4 times/year	12	7.64
	3 times/year	2	1.27
	2 times/year	19	12.10
	1 time/year	10	6.37
	+ than once/month	6	3.81
	All visits	57	36.31
Inspection	Yes	123	78.34
	No	34	21.66
Risk assessment	Yes	126	80.25
	No	31	19.75
Self-care education	Never	3	1.91
	Rare	6	3.82
	Occasionally	16	10.19
	Frequently	55	35.03
	Very frequently	77	49.04
Skills, knowledge, and environment check	Never	9	5.73
	Rare	16	10.19
	Occasionally	34	21.66
	Frequently	66	42.04
	Very frequently	32	20.38

**Table 5:** Conducting workshops. (n = 127).

Interventions	Value	Frequency (n)	Percentage (%)
Conducts workshops (n = 157)	Yes	30	19.11
	No	127	80.99
Reasons for not conducting workshops (n = 127)	No response	32	25.20
	Time	35	27.56
	Training	1	0.79
	Type of patient	14	11.02
	No service	16	12.60
	Individual	9	7.09
	Resources	17	13.39
	Time + individual	1	0.79
	Time + resources	2	1.57

**Table 6:** Chi^2^ (χ^2^) analysis between the characteristics of the population and the activities carried out.

Interventions	Gender		Age		Years in Service		Work Centre		Podiatrist		NRI		Specific Training		Quota DM		Treated Ulcers	
	χ^2^	*p*-Value	χ^2^	*p*-Value	χ^2^	*p*-Value	χ^2^	*p*-Value	χ^2^	*p*-Value	χ^2^	*p*-Value	χ^2^	*p*-Value	χ^2^	*p*-Value	χ^2^	*p*-Value
Barefoot exploration	5.49	0.064	18.73	0.016	13.83	0.086	2.47	0.291	0.52	0.770	0.71	0.703	1.01	0.603	5.53	0.853	5.66	0.933
Inspection	0.7	0.874	22.48	0.032	11.62	0.477	0.42	0.809	1.60	0.659	6.32	0.097	5.62	0.132	7.36	0.691	18.38	0.431
Risk assessment	0.64	0.424	3.72	0.445	2.7	0.610	28.1	0.000	0.012	0.893	1.37	0.241	7.7	0.006	55.83	0.000	2.3	0.891
Self-care education	2.54	0.281	4.74	0.785	4.18	0.840	0.12	0.994	1.06	0.588	0.02	0.992	4.7	0.096	6.74	0.750	14.35	0.279
Skills and knowledge	0.07	0.996	10.83	0.211	11.88	0.157	2.24	0.326	1.90	0.387	1.88	0.390	2.44	0.295	13.9	0.178	7.9	0.793
Workshops	0.2	0.656	0.55	0.968	0.96	0.916	0.14	0.706	2.28	0.131	0.25	0.617	0.97	0.324	5.97	0.310	0.29	0.218

χ^2^: Bivariate analysis between two qualitative variables (Chi-squared distribution); the relationship between the characteristics of the population and the different activities evaluated. *p*-Value: statistical significance at a *p*-value < 0.05, representing 95% confidence.
